# Multi-Channel Multi-Scale Convolution Attention Variational Autoencoder (MCA-VAE): An Interpretable Anomaly Detection Algorithm Based on Variational Autoencoder

**DOI:** 10.3390/s24165316

**Published:** 2024-08-16

**Authors:** Jingwen Liu, Yuchen Huang, Dizhi Wu, Yuchen Yang, Yanru Chen, Liangyin Chen, Yuanyuan Zhang

**Affiliations:** 1 College of Computer Science, Sichuan University, Chengdu 610065, China; liujw@stu.scu.edu.cn (J.L.); yuchen_huang@stu.scu.edu.cn (Y.H.); wudizhi1234@163.com (D.W.); chenyanru@scu.edu.cn (Y.C.); chenliangyin@scu.edu.cn (L.C.); 2 College of Computer Science, East China Normal University, Shanghai 200062, China; 10225102401@stu.ecnu.edu.cn; 3 Institude for Industrial Internet Research, Sichuan University, Chengdu 610065, China

**Keywords:** industrial control systems, anomaly detection, variational autoencoder, anomaly interpretation

## Abstract

With the rapid development of industry, the risks factories face are increasing. Therefore, the anomaly detection algorithms deployed in factories need to have high accuracy, and they need to be able to promptly discover and locate the specific equipment causing the anomaly to restore the regular operation of the abnormal equipment. However, the neural network models currently deployed in factories cannot effectively capture both temporal features within dimensions and relationship features between dimensions; some algorithms that consider both types of features lack interpretability. Therefore, we propose a high-precision, interpretable anomaly detection algorithm based on variational autoencoder (VAE). We use a multi-scale local weight-sharing convolutional neural network structure to fully extract the temporal features within each dimension of the multi-dimensional time series. Then, we model the features from various aspects through multiple attention heads, extracting the relationship features between dimensions. We map the attention output results to the latent space distribution of the VAE and propose an optimization method to improve the reconstruction performance of the VAE, detecting anomalies through reconstruction errors. Regarding anomaly interpretability, we utilize the VAE probability distribution characteristics, decompose the obtained joint probability density into conditional probabilities on each dimension, and calculate the anomaly score, which provides helpful value for technicians. Experimental results show that our algorithm performed best in terms of F1 score and AUC value. The AUC value for anomaly detection is 0.982, and the F1 score is 0.905, which is 4% higher than the best-performing baseline algorithm, Transformer with a Discriminator for Anomaly Detection (TDAD). It also provides accurate anomaly interpretation capability.

## 1. Introduction

In recent years, with the increasing focus on industrial control systems and the rapid development of IoT technologies, more and more novel networking technologies have been integrated into industrial control systems to enhance equipment monitoring and improve production processes. However, this growing connectivity to external networks also means that industrial control systems face higher risks [1,2]. Researchers often equip industrial devices with high-precision, high-sampling-frequency sensors to obtain comprehensive operational data. Yet, in factory environments, anomalous data can propagate rapidly and have significant consequences if ignored or not promptly detected. For instance, if a critical sensor on a production line experiences abnormal values due to external attacks and is not promptly restored, it may lead to localized failures that quickly spread throughout the assembly line, resulting in downtime and cost losses. Given the large scale of industrial facilities, the consequences of such failures are often difficult to estimate. Therefore, deploying anomaly detection algorithms within industrial control systems is essential.

Currently, high-precision anomaly detection algorithms [3,4,5] can be divided into three categories. For example, LSTM and Non-parametric Dynamic Thresholding (LSTM-NDT) proposed by Hundman et al. [6] models each dimension’s time series variables separately, then takes the multi-dimensional variables at historical moments as input, predicts the multi-dimensional variable values at the next moment, and calculates the prediction error. Multi-Scale Convolutional Recurrent Encoder–Decoder, proposed by Zhang et al. [7], uses a feature matrix to characterize time series and then applies an encoder–decoder structure to learn to reconstruct data patterns for anomaly detection. These algorithms capture the time dependence within dimensions well but ignore the correlation between dimensions, so the accuracy of anomaly detection for sensor data with high correlation in industrial control scenarios is not high. Other reconstruction methods, such as the OmniAnomaly algorithm proposed by Su et al. [8], use Simple Recurrent Neural Network and multiplicative attention mechanism technology to mine variables’ correlations. Multi-variate Anomaly Detection with Generative Adversarial Network (MAD-GAN) proposed by Li et al. [9] adds attention mechanisms between the generator and discriminator, allowing the model to focus on different dimensions when generating or discriminating time series data. Although these models capture correlation features better in the above ways, they lack the process of modeling each dimension along the time series. Hence, the detection effect of time-dependent anomalies is poor. The third category of algorithms, such as LSTM-based VAE-GAN proposed by Niu et al. [10], jointly trains encoders, generators, and discriminators, modeling time dependence and relationship correlation dependence at the same time to simultaneously utilize the mapping ability of the encoder and the discrimination ability of the discriminator. However, these methods cannot indicate or explain the reasons or factors behind data being identified as anomalies [11]. We have found that for complex high-dimensional time series data, most existing models cannot simultaneously capture intra-dimensional temporal and inter-dimensional relational features. Large-scale factories generate multi-dimensional time series data that often contain both types of features, so the accuracy of current anomaly detection algorithms needs improvement. For example, in large manufacturing plants, multiple sensors and actuators operate simultaneously, generating vast multi-dimensional time series data. Existing models might detect anomalies of a single type but fail to simultaneously identify both intra-dimensional and inter-dimensional anomalies. Furthermore, some complex algorithms, although capable of considering both intra-dimensional and inter-dimensional features, often lack interpretability. These models might indicate that an anomaly occurs at a specific time but struggle to identify the faulty sensor or device. Due to this lack of interpretability, these algorithms are difficult to trust and widely adopted in practical industrial applications. Overall, current multi-variate time series anomaly detection methods cannot effectively capture temporal features within dimensions and relational features between dimensions; some algorithms that consider both types of features lack interpretability.

To solve the above problems, we propose an interpretable anomaly detection algorithm based on variational autoencoder (VAE), MCA-VAE (Multi-Channel Multi-Scale Convolution Attention Variational Autoencoder). MCA-VAE can simultaneously extract both intra-dimensional and inter-dimensional features, achieving good anomaly detection performance. Additionally, it can explain anomalies by identifying the sensors most likely to cause the anomalies, facilitating prompt maintenance by researchers and significantly improving industrial production efficiency. To address the issue of existing models not effectively extracting both types of features simultaneously, we use a multi-scale weight-sharing convolutional network to capture the temporal features within each dimension and a multi-head attention mechanism to capture the relationship features between different dimensions. Additionally, we employ the reparameterization method to separate the sampling process from differentiable operations, accelerating the convergence speed. We detect anomalies based on the reconstruction error of VAE. To address the issue of anomaly interpretability, we utilize the probability distribution of the variational autoencoder, which allows us to obtain the anomaly scores of each sensor quickly. The ranking table of anomaly scores obtained after sorting can help engineers rapidly locate anomalies. Experimental results demonstrate that our algorithm attains an AUC value of 0.982 and an F1 score of 0.905 in anomaly detection, surpassing the best-performing baseline algorithm, Transformer with a Discriminator for Anomaly Detection (TDAD), by 4%. Additionally, it offers accurate anomaly interpretation capability.

In summary, the main contributions of this manuscript are as follows:

(1) To address the challenge of neural network models deployed in factories that struggle to capture intra-dimensional temporal and inter-dimensional relational features simultaneously, we propose a high-precision anomaly detection algorithm based on VAE. Specifically, we propose a multi-scale local weight-sharing convolutional neural network to extract intra-dimensional features and utilize multi-head attention mechanisms to capture inter-dimensional features fully. Additionally, we assign greater weight to the reconstruction error to enhance the VAE reconstruction performance. We use reparameterization techniques to accelerate convergence and detect anomalies through reconstruction errors, achieving high-precision anomaly detection.

(2) To address the challenge of existing methods that do not perform well in locating anomalies within industrial control systems, we utilize an improved VAE with a reconstruction probability model to explain and pinpoint anomalies. The probabilistic distribution of the VAE allows us to obtain anomaly scores for each sensor easily, and the resulting ranked table of anomaly scores facilitates the rapid localization of anomalies by researchers.

(3) The experimental results show that on the industrial control dataset, MCA-VAE performs best regarding F1 score and AUC value. MCA-VAE achieves an AUC value of 0.982 and an F1 score of 0.905, which is 4% higher than the best baseline algorithm, TDAD. Additionally, based on reconstruction probability, the sensors with higher anomaly scores match the sensors that generate anomalies, providing accurate anomaly explanation capabilities.

The remainder of this paper is organized as follows. Section 2 examines related work. Section 3 provides a detailed description of the algorithm proposed in this paper. Section 4 analyzes the performance of the algorithm proposed in this paper in different aspects through experiments. Section 5 concludes this paper.

## 2. Related Work

Large factories generate high-dimensional and complex data from numerous devices and sensors, making traditional statistical methods less effective for multi-variable time series anomaly detection. Neural network models, with their non-linear modeling capabilities, adaptability, and robustness, are increasingly used to address these challenges [12]. Recurrent neural networks like Long Short-Term Memory (LSTM) and Gated Recurrent Unit (GRU) are common choices due to their proficiency with time series data. Newer models like autoencoders, variational autoencoders, and generative adversarial networks also play a significant role in anomaly detection, as they can learn data distribution rules and feature information through unsupervised learning. Meanwhile, anomaly interpretability is very important because it can provide detailed information about the anomaly detection results, thus helping engineers and technicians better understand the root cause of the problem and take corresponding measures. For example, suppose the anomaly detection result shows that a particular device has an anomaly but does not provide any interpretable information. In that case, the engineer will have difficulty solving the problem. However, the anomaly detection result provides more information about the problem, such as which specific device has an anomaly, or when the anomaly occurs. In that case, the engineer can identify and solve the problem faster. At present, there are fewer research achievements related to interpretable multi-variable anomaly detection algorithms.

Li et al. [9] built a GAN model using a generator and discriminator to adaptively detect anomalies in multi-dimensional time series. The shortcoming is that, like other GAN-based models, MAD-GAN also faces the instability problem of GAN. Zong et al. [13] proposed a deep autoencoding Gaussian mixture model, using a deep autoencoder to extract low-dimensional representations and further extracting dimension correlation with a Gaussian mixture model while jointly optimizing parameters with an estimation network. Zhang et al. [14] proposed a graph structure change-based anomaly detection on multi-variate time series. Tuli et al. [15] proposed the Transformer-based Anomaly Detection Model (TranAD), a deep transformer network-based anomaly detection and diagnosis model which used attention-based sequence encoders to swiftly perform inference with the knowledge of the broader temporal trends in the data. Although they better capture the correlation features, they lack the process of modeling each dimension along the time series, resulting in poor detection performance for time-dependent anomalies. Liang et al. [16] introduced the multi-time scale deep convolutional generative adversarial network framework for anomaly detection in industrial time series. It converts multi-variate time series into multi-channel matrices using sliding window cross-correlation and a forgetting mechanism to reduce false alarms. However, it captures the time dependency within dimensions well but ignores the correlation between dimensions, resulting in low accuracy for the anomaly detection of highly correlated sensor data in industrial control scenarios. Niu et al. [10] proposed a VAE-GAN that jointly trains encoders, generators, and discriminators based on LSTM, modeling time dependence and relationship correlation dependence at the same time, to simultaneously utilize the mapping ability of the encoder and the discrimination ability of the discriminator. Nizam et al.’s [17] proposed framework is based on a convolutional neural network (CNN) and a two-stage LSTM-based autoencoder (AE). However, its ability to model time dependencies is weak, and it lacks interpretability.

Rajendran et al. [18] proposed a radio spectrum anomaly detection method based on Adversarial Autoencoder (AAE), which can achieve anomaly detection and localization through Power Spectral Density (PSD) data. Although the interpretability accuracy of this model is high, it is challenging to obtain labeled data in the factory environment, and semi-supervised methods cannot work well. Deng et al. [19] used graph-related methods to learn the relationship between sensors and used this relationship to detect and explain deviations in learning patterns. However, this method can only identify single-point anomalies, and it cannot locate well for regional anomalies, such as multiple sensors showing anomalies at the same time in a certain period. Wang et al. [20] aimed to detect anomaly points in multi-variate time series data. They determined the anomaly values by calculating the distance of the subsequence. They used a dynamic programming algorithm to allocate the anomaly points to the position in the original sequence. The disadvantage is that this method cannot handle noise data, and it cannot handle situations where the anomaly sequences overlap; that is, the starting position of one anomaly sequence may overlap with the ending position of another anomaly sequence.

We found that current methods, although using neural networks to model multi-dimensional variables, often fail to simultaneously consider the temporal features within individual dimensions and the interaction between different dimensions. This oversight may lead to the neglect of useful features and a decrease in detection accuracy. Additionally, some combination models that consider both types of features lack interpretability, making it challenging to provide precise sensor anomaly localization [21]. Furthermore, existing anomaly explanation algorithms face difficulties when applied to large-scale factory environments for multi-variable anomaly detection and localization tasks. We propose an interpretable high-precision unsupervised anomaly detection algorithm in response to the two issues above. Our approach models features within and across dimensions, leveraging an improved VAE to achieve accurate anomaly detection while assisting engineers in quickly locating anomalous sensors.

## 3. Methodology

To illustrate the necessity of modeling different features in time series data, we first review the anomalies in univariate and multi-variate time series data. We describe the characteristics of three types of anomalies in multi-variate time series data. We then define the problem of multi-variate time series anomaly detection and anomaly interpretation. Next, we describe our method’s specific process and algorithmic network structure. The following sections introduce our proposed multi-scale local weight-sharing convolutional neural network model for extracting temporal features within dimensions and the multi-head attention mechanism for extracting relational features between dimensions. We then explain how we improve the reconstruction performance and convergence speed of the VAE. Finally, we describe how to use the reconstruction probability of the variational autoencoder to interpret anomalies.

### 3.1. Aim and Scope

#### 3.1.1. Types of Anomalies in Multi-Variate Time Series

**Figure 1:**
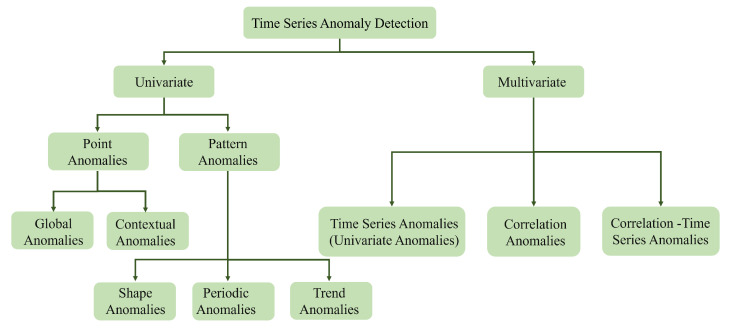
Classification chart for time series detection.

Time series anomaly detection can be divided into univariate and multi-variate based on the detection target as shown in Figure 1. The anomaly types of univariate time series can be divided into two major categories, namely, point anomaly and pattern anomaly. A series of continuous points causes pattern anomalies, which can be further divided into periodic anomalies, shape anomalies, and trend anomalies. The anomaly types of multi-variate time series can be divided into three categories. In addition to including the most common anomaly types of univariate (time series anomaly), there are two unique anomaly types, which are the correlation anomaly and correlation–time anomaly. The correlation anomaly indicates that the relationship between multiple indicators has undergone a sudden change or violated the historical relationship. This type of anomaly is usually caused by a sudden event of one or more variables, causing the relationship between this variable and other variables to change. The correlation–time anomaly includes both the correlation anomaly and time anomaly, and it is the most accessible type of anomaly to detect.

**Figure 2:**
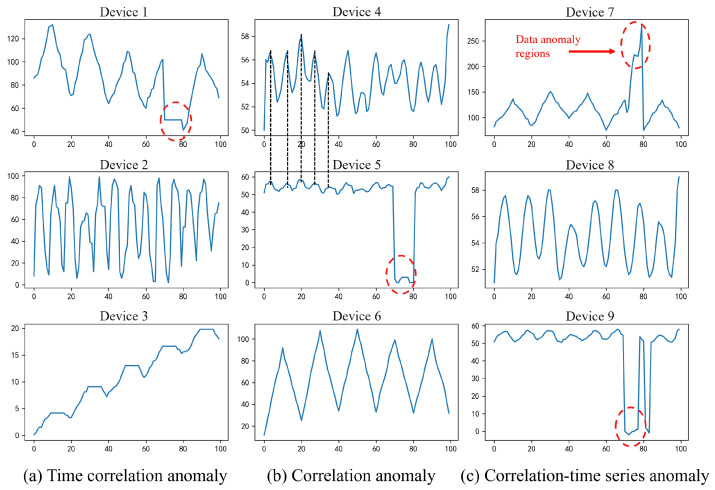
Diagram of sensor anomaly types.

To better describe the correlation anomaly and time anomaly in multi-variate time series, we use a set of real sensor data as an example. SWaT (Secure Water Treatment) [22] is essential for research in the design of secure Cyber–Physical Systems. It is a scaled-down version of a real-world industrial water treatment plant. Researchers can use this dataset to develop and evaluate security solutions for industrial control systems. The data come from a section of 9 sensor devices in the SWaT, with an effective duration of 11 min. The types of sensors include pressure, water level, etc. The sensor data are shown in Figure 2.

Figure 2a shows the most common type of time series correlation anomaly. Sensors 2 and 3 are normal during the detection time. However, the data of device 1 in the red area have undergone a sudden change compared to their own data’s linear pattern of rising and falling over some time, becoming a horizontal line. This significant deviation from its corresponding historical normal pattern violates the time correlation. Figure 2b shows the type of correlation anomaly. By observing the data of sensors 4 and 5, it can be found that the historical pattern shows a positive correlation in the non-red area. Still, device 5 violates this pattern in the red area, violating the correlation. The detection of this type of anomaly requires a clear understanding of the relationships between multi-dimensional data, which poses a significant challenge to algorithm research. Figure 2c shows the correlation–time kind of anomaly. It can be seen that for sensors 7 and 9, the historical data show a regular rise and fall pattern. Still, in the red area, the value shows violent fluctuations, indicating that sensors 7 and 9 violate the time correlation. Secondly, by observing sensors 8 and 9, it can be seen that there is a clear positive correlation in the non-red area. Still, sensor 9 maintains a horizontal line value without change in the red area, indicating that sensor 9 also has a fault. That is to say, in the type of correlation–time anomaly, both time dependency and relationship dependency are violated, so this type is most often easier to detect. Whether severe or subtle, each type of anomaly may indicate potential problems in the system. Therefore, the goal of the multi-variate anomaly detection system should be to detect as many of the three anomalies as possible. For this, the model design should be able to simultaneously capture the time features within the sensor at the same moment, as well as the relationship features between the sensor and other sensors.

We found that current detection models either focus on modeling each dimension of time series data, ignoring the inter-dimension correlation in time series data or use complex structures to extract inter-dimension correlation but lack a detailed modeling process for the time dependency of each dimension datum. Therefore, in order to achieve better anomaly detection accuracy, it is necessary to model both the time dependency within dimensions and the correlation between dimensions simultaneously.

#### 3.1.2. Definition of the Problem

**Figure 3:**
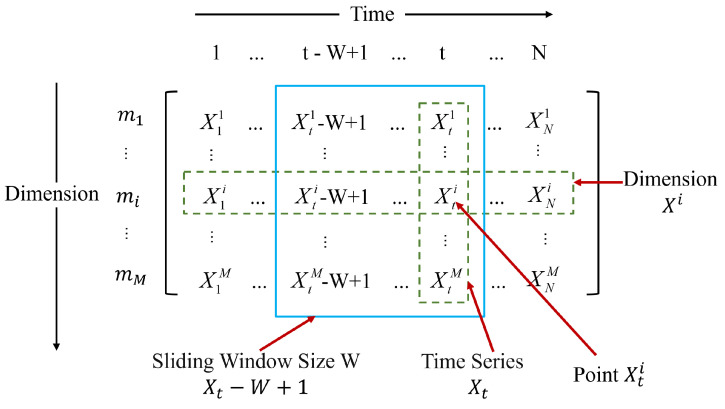
Multi-variate time series problem definition diagram.

Figure 3 represents a set of multi-variable time series (MTS) data. The dataset, denoted as $X=\left\{X_{1},\ldots ,X_{N}\right\}$, $X\in R^{M\times N}$, contains continuous observations with equal interval sampling, where *M* and *N* are the number of dimensions and the length of the data, respectively. In each row, $X^{i}$ represents a dimension, and in each column, $X_{t}$ is an observation. Taking the data from a Web application server as an example, dimensions include CPU utilization, memory utilization, the number of TCP connections actively opened, the number of packets transmitted per second, etc. The entire entity (i.e., the multi-variate time series data) characterizes the state of the server. Multi-variate time series data typically have time correlation within each dimension (for example, the periodicity of CPU utilization), as well as the correlation between different dimensions (for example, the positive correlation between the number of packets transmitted per second, the number of TCP connections actively opened, and CPU utilization). To fully incorporate contextual information, we apply a sliding window of length *W* on the MTS to calculate the anomaly results. This transforms the MTS into a set of windowed samples, which are then used as inputs to the model. Under normal circumstances, these sensor values may fluctuate within a certain range. However, if at a specific time point, the value of a particular sensor significantly deviates from the expected range—for example, a sudden increase in temperature or an unexpected pressure drop—then that data point could be considered an anomaly. Anomaly detection aims to determine whether the observed result $X_{t}$ is abnormal. Anomaly interpretation is achieved through localization, that is, finding a set of dimension values ($\left\{X^{1},\ldots ,X^{i}\right\}$, i<=M) that are the most anomalous for each detected entity anomaly $X_{t}$.

### 3.2. Algorithm Structure

#### 3.2.1. Overall Architecture

**Figure 4:**
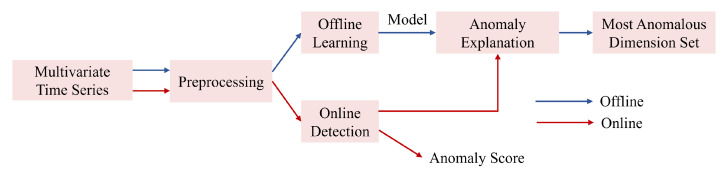
MCA-VAE overall architecture diagram.

Our method as shown in Figure 4 consists of two parts: offline training and online detection. Data preprocessing is a module shared by these two parts. During data preprocessing, the dataset is standardized and then divided into multiple subsequences by a sliding window. The preprocessed multi-variate time series data are sent to the model training module for training in order to establish a model that can capture the normal patterns in the multi-variate time series. The online detection module stores the trained model. Then, the preprocessed new data window (for example, $X_{t}$ at time *t*) is sent to the online detection module to obtain its anomaly score. If the anomaly score of $X_{t}$ is higher than the anomaly threshold, $X_{t}$ will be classified as an anomaly; otherwise, it is considered normal. If $X_{t}$ is detected as an anomaly, it is interpreted by estimating and ranking the contribution (i.e., reconstruction probability) of each dimension.

#### 3.2.2. Network Structure

**Figure 5:**
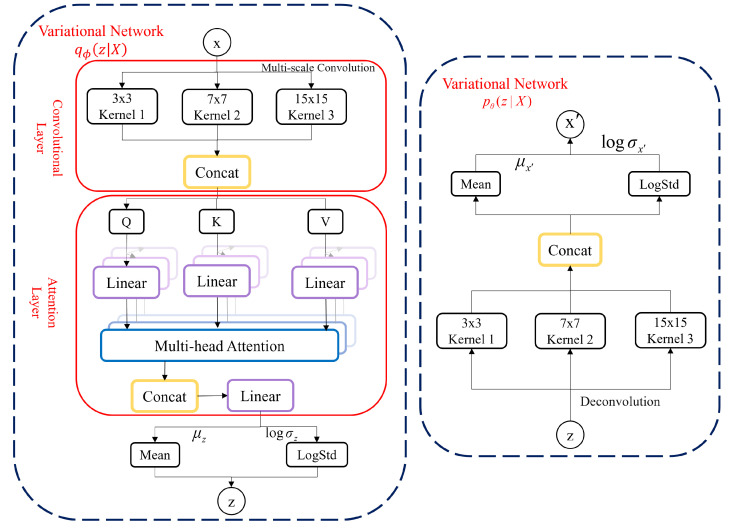
MCA-VAE network architecture model diagram.

Our model is composed of three parts as shown in Figure 5.

1. Multi-Scale Weight Sharing Convolutional Network: The multi-scale convolutional neural network can effectively capture features at different time scales. Smaller convolution kernels can capture short-term time series features, while larger convolution kernels can capture long-term time series features. At the same time, the proposed multi-channel local weight-sharing method can reduce the number of parameters as much as possible while ensuring accuracy.
2. Multi-Head Attention Mechanism: The multi-head attention mechanism can flexibly pay attention to the mutual influence between different data dimensions, thereby helping the model capture the relationship features between dimensions.
3. Improved Variational Autoencoder (VAE): The VAE is used to model the output of the attention from a global perspective. The proposed KL divergence optimization method can make the VAE model training achieve better feature extraction results. In addition, the probability distribution of the VAE model is used for anomaly interpretation.

### 3.3. Multi-Scale Weight Sharing Convolutional Network

Most of the data in factories are multi-variate time series. Traditional 1D CNN models are only suitable for single-channel time series data and cannot fully exploit the information from multiple correlated channels. Multi-channel 1D CNN models use multiple independent convolution kernels to perform convolution operations on each channel and then merge the convolution results of each channel. This approach can simultaneously extract temporal features from multiple dimensions and fully utilize the information from multiple channels through the merging operation, thereby enhancing the performance and accuracy of the model. To achieve this, the algorithm must create multi-channel kernels *k* to process each input data channel independently. The convolution operation of multi-channel kernel *k* on multi-channel data is shown in Equation (1):

(1) $$\begin{array}{c} St=\left(x⊗k\right)\left[t\right]=\sum_{c=1}^{n_{c}}\sum_{i=1}^{n_{k}}X_{c,t-i+1}K_{c,i} \end{array}$$

where $n_{c}$ is the number of channels in the data, and $X_{c}$ is the measurement of the *h*-th channel at time step t−i+1. The convolution on the multi-channel data is almost similar to that on a single channel; the difference is the additional summation on all channels. However, we note that in multi-channel convolution, a convolution kernel is set for each channel, and the number of weights for each convolution kernel will be more, making the model parameters large, slowing down the training speed, and increasing the risk of overfitting.

In addition, existing models use 1D CNN to process time series data. A fixed-size convolution kernel is commonly used to extract features from each subsequence and capture time and space information. We note that since 1D CNN can only capture local temporal features, for time series data with long-term dependencies, 1D CNN may not capture key information. Therefore, a fixed-size convolution kernel can only capture features of a specific scale and cannot adapt to different time scales.

Therefore, we propose an improved multi-scale weight-sharing convolution method. We propose a multi-channel local weight-sharing method to balance many parameters and the effect of feature extraction. We also propose a multi-scale convolution kernel method to solve the problem of inability to adapt to different time scales.

#### 3.3.1. Implementation of Multi-Channel Local Weights

First, we introduce the multi-channel local weight-sharing method. Weight sharing refers to using the same parameters for convolution kernels at different positions in a multi-channel convolutional neural network. This method can significantly reduce the number of parameters in the neural network, as the number of parameters for each convolution kernel can be compressed to the same number as a single convolution kernel. For a multi-channel convolutional neural network, each convolution kernel needs to perform convolution operations with multiple channels, so more parameters are required. However, if the weight-sharing method is used, the same position convolution kernels on all channels can use the same parameters, thereby greatly reducing the number of parameters. This can effectively solve the problem of large parameter volume in multi-channel convolutional neural networks.

Specifically, suppose a convolution layer contains *k* convolution kernels; each convolution kernel size is h×w, the number of input channels is $c_{in}$, and the number of output channels is $c_{out}$. If the non-weight sharing method is used, the number of parameters for this layer is $k\times h\times w\times c_{in}\times c_{out}$. But if the weight-sharing method is used, the number of parameters for this layer is only k×h×w. Therefore, weight sharing can greatly reduce the number of parameters, thereby reducing the model’s complexity and improving the model’s training speed and generalization performance.

We notice that the global sharing method has a problem of insufficient learning because the convolution kernels all use the same weight matrix. Although it greatly reduces the number of parameters, it causes channels with large differences in original data types to have poor learning effects. Suppose there are very different data characteristics between dimensions in the input data. In that case, the convolution kernel using global sharing will not be able to adapt to all features fully, causing the model to be insufficiently learned and unable to extract features accurately.

In response to this problem, we propose a local weight-sharing method. Local free weight sharing refers to weight sharing only in specific areas so that each area can freely adjust the weights to better adapt to the features of the data. The traditional global weight sharing is achieved by sharing a parameter matrix. In contrast, local free weight sharing is achieved by decomposing the weight matrix into multiple local weight matrices, and each local weight matrix can be adjusted independently. This structure reduces the number of parameters while ensuring good feature extraction capabilities. The calculation method for traditional global weight sharing is shown in Equation (2). The local free weight sharing formula is shown as Equation (3):

(2) $$\begin{array}{c} y_{i,j,k}=\sum_{m,n,l}W_{m,n,l,k}X_{i+m,j+n,l} \end{array}$$

(3) $$\begin{array}{c} y_{i,j,k}=\sum_{m,n,l}W_{m,n,l,k}^{\left(i,j\right)}X_{i+m,j+n,l} \end{array}$$

where $W_{m,n,l,k}^{\left(i,j\right)}$ represents the weight in the local weight matrix at the coordinate (i,j). To fully utilize the advantages of multi-channel free weight sharing, we propose to analyze the correlation between data and first use the same local weight matrix for multi-dimensional data with strong correlation. This can reduce the number of parameters while preserving the feature extraction capability. For example, for the dataset we use, we observe that the data characteristics of AIT502, UV501, AIT501, and AIT502 are all large jumps, with a period of 2000 time points, and have obvious similarities, so we can use the same local free weight sharing for these channels to optimize.

#### 3.3.2. Implementation of the Multi-Scale Convolutional Network

To address the issue that fixed convolutional kernels can only capture features of a specific scale, we propose a multi-scale convolutional kernel to extract highly expressive latent features of different time scales from the dataset. We construct a convolutional layer with three parallel paths, each with different kernel sizes, and each convolutional layer uses the multi-channel convolution method described earlier.

**Figure 6:**
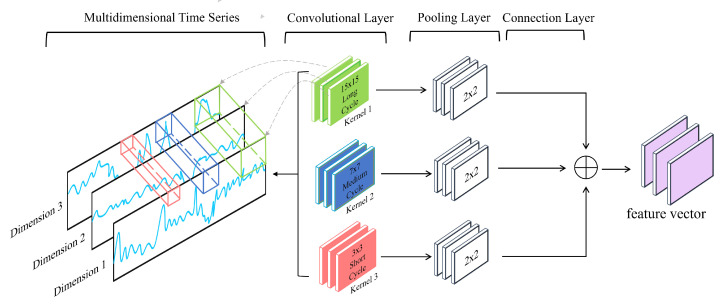
Multi-scale convolutional block model diagram.

Factory data contain short-term regularities (considering the instant when a product enters or leaves a process on a single sensor scale), medium-term patterns (considering the production process occurring within a single process), and long-term changes (considering the entire cycle from one product to the next, including transportation, production, and inspection). To cover these three situations, we choose short cycles of size 3, medium cycles of size 7, and long cycles of size 15 for the 1D convolutional layer (Conv 1D) convolutional kernel to slide in the time domain. This way, the multi-scale convolution module can simultaneously extract data trends and hidden interactions in the sequence with periods of 3, 7, and 15 time units. Furthermore, we know that the one-dimensional convolutional kernel’s size will affect the network’s learning effect. For example, when the convolutional kernel is small, it is conducive to detecting point anomalies because the duration of point anomalies is concise (caused by a single time point), and it can fully capture local feature information. When the convolutional kernel is large, it is conducive to capturing longer types of anomalies, such as pattern anomalies, because the duration of pattern anomalies is very long (caused by a series of continuous points). Therefore, our multi-scale convolutional kernel design can also consider the advantages of both large and small kernels, helping the model learn at different scales. Finally, the feature vectors obtained after convolution and pooling are connected to form a new global feature vector matrix. This way, the feature maps extracted from the three different cycles will be fused, and the subsequent attention mechanism will adaptively extract helpful information from these hierarchical features. Our multi-scale convolution module is shown in Figure 6.

After extracting features using convolutional layers of different scales, we perform feature selection and downsampling to reduce the spatial dimension of the data and retain the most significant features. We use max pooling to perform downsampling operations on the feature maps as shown in Equation (4):

(4) $$\begin{array}{c} {\hat{c}}_{k}=max\left\{c_{k}\right\} \end{array}$$

where $c_{k}$ represents the feature map extracted using a kernel of size *k*, and ${\hat{c}}_{k}$ is the feature map after sampling. The feature vectors obtained after the convolution and pooling operations are represented by ${\hat{C}}_{3}$, ${\hat{C}}_{7}$, and ${\hat{C}}_{15}$. These three feature vectors are connected into a global feature vector matrix T as shown in Equation (5). This global feature vector matrix T will be input into the multi-head attention mechanism for further processing:

(5) $$\begin{array}{c} T=\hat{C_{3}}⊕\hat{C_{7}}⊕\hat{C_{15}} \end{array}$$

### 3.4. Multi-Head Attention Mechanism

To capture the correlation features between dimensions, we use a multi-head attention mechanism to further extract useful information from the convolutional network obtained in the previous stage. Although the attention mechanism has achieved good results in different fields, the single-head attention mechanism has problems such as large computation and easy overfitting when facing high-dimensional data. The advantage of the multi-head attention mechanism is that it can flexibly combine and aggregate the input sequence, making it more suitable for modeling and processing long and multi-dimensional sequences. At the same time, the multi-head attention mechanism can pay attention to different time steps, improving the robustness and stability of the model.

Using the multi-head attention mechanism to extract the correlation between dimensions, we first initialize the matrices Q, K, and V from the global feature vector matrix *T* obtained in the previous convolutional network. These three matrices serve as key parameters for the single-head attention mechanism. The main idea of the single-head attention mechanism is the Scaled Dot-product Attention (SDA), which first calculates the similarity by solving the dot product of Q and K and then divides by $\sqrt{d_{k}}$ ($d_{k}$ is the dimension of matrix K) to prevent the dot product calculation result from being too large. Then, it normalizes the result through the Softmax function and multiplies it by the matrix V to obtain the attention expression. The calculation method of SDA is shown in Equation (6):

(6) $$\begin{array}{c} SDA\left(Q,K,V\right)=Softmax\left(\frac{QK^{T}}{\sqrt{d_{k}}}\right) \end{array}$$

**Figure 7:**
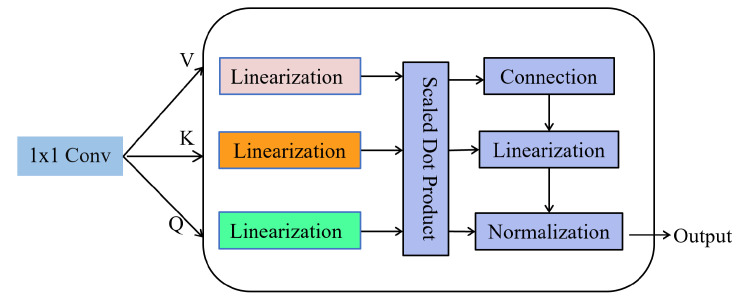
Multi-head attention mechanism model.

Unlike the standard attention mechanism, the multi-head attention mechanism introduces multiple combinations of queries, keys, and values, thereby enhancing the attention mechanism’s ability to extract different information from the input sequence. The idea is to use different parameters $W_{i}^{Q}$, $W_{i}^{K}$, and $W_{i}^{V}$ to perform linear transformations on matrices Q, K, and V, in turn, inputs the linear transformation results into SDA. The head expresses the calculation result as shown in Equation (7). The calculation results from $head_{i}$ to $head_{h}$ are concatenated into a matrix, and multiplying by the parameter *W* completes the final linear transformation, resulting in the final output of the multi-head attention mechanism as shown in Equation (8). The training process of our model after introducing the attention mechanism is shown in Figure 7:

(7) $$\begin{array}{c} head_{i}=SDA\left(QW_{i}^{Q},KW_{i}^{K},VW_{i}^{V}\right) \end{array}$$

(8) $$\begin{array}{c} Head=MultiHead\left(Q,K,V\right)=Concat\left(head_{1},\ldots ,head_{h}\right)W \end{array}$$

### 3.5. Improved Variational Autoencoder

To further capture data features from a global perspective and provide model interpretability, we use VAE to map the output of the previous step into the latent space.

To model the information of multiple variables from a global perspective and better capture the dependencies between different dimensions, we propose mapping the output of the attention mechanism into the latent space of the VAE model. The stochastic nature of VAE allows it to learn data features from the latent space, including the distribution and change patterns of regular data, thereby better adapting to complex data distributions. At the same time, the reconstruction probability of VAE can provide interpretability for anomalies.

However, we notice that when the ordinary VAE uses optimization methods such as stochastic gradient descent (SGD) to train the VAE, the encoder tends to map each input to a fixed point in its latent distribution. This makes the KL divergence term in the VAE constant and no longer related to the latent variables; thus, it cannot be effectively optimized. To solve this problem, we propose an improved VAE method with a modified Evidence Lower Bound (ELBO) expression and reparameterization optimization.

#### Implementation of Improved Variational Autoencoder

The basic theory of VAE [23] is as follows: suppose x is the input data, and z is the latent variable. VAE aims to learn a conditional distribution $p_{\theta }\left(z|X\right)$ so that a given x can be sampled from the latent space z and generate new samples similar to x. To achieve this goal, VAE first maps the input data x to the latent space z, obtaining the encoder $q_{\phi }\left(z|X\right)$. Then, $q_{\phi }\left(z|X\right)$ samples a latent variable z from it and decodes it into data $X^{\prime }$ similar to X, obtaining the decoder $p_{\theta }\left(X|z\right)$. To ensure that the data $X^{\prime }$ generated by the decoder are similar to the input data X, VAE introduces a reconstruction error term, representing the difference between X and $X^{\prime }$. At the same time, to give the learned latent space z a specific structure and continuity, VAE also introduces a regularization term, the prior distribution $p\left(z\right)$ of the latent variable z.

Since directly calculating $p_{\theta }\left(X|z\right)$ is very difficult, VAE uses a technique called Variational Inference to train the model to achieve this goal. Specifically, the loss function (ELBO) of VAE can be expressed as shown in Equation (9):

(9) $$\begin{array}{c} ELBO=E_{q_{\phi }\left(z|X\right)}\left[\log p_{\theta }\left(X|z\right)\right]-KL\left(q_{\phi }\left(z|X\right)\|p\left(z\right)\right) \end{array}$$

Here, $E_{q_{\phi }\left(z|X\right)}$ represents the expectation of *z* given the input *X*. $KL\left(q_{\phi }\left(z|X\right)|p\left(z\right)\right)$ represents the KL divergence between the posterior distribution $q_{\phi }\left(z|X\right)$ and the prior distribution $p\left(z\right)$. By minimizing this loss function, VAE can learn the latent distribution of the data and generate new samples from it. However, we notice that in practical applications, if the weight of the KL divergence term is too large or there is a significant difference between the distribution of the dataset and the prior distribution, the model tends to map the input samples to a small area in the latent space, ignoring the diversity between samples. Specifically, the KL divergence term in the objective function of VAE penalizes the difference between the distribution in the latent space and the prior distribution. To minimize the KL divergence, the model will strive to make the learned latent variable distribution close to the prior distribution, which usually causes the sample points in the latent space to cluster together, forming a compact cluster. The samples generated by the generator network by decoding these sample points are similar or repetitive. The model maps the input samples to the area close to the prior distribution to minimize the KL divergence term rather than exploring a broader distribution in the latent space. To solve this problem, we optimize the first term of ELBO (i.e., the reconstruction error) and give it more weight. The modified ELBO is defined as shown in Equation (10). Here, β is a hyperparameter used to control the relative importance of the reconstruction and KL divergence errors. By adjusting the value of β, the model’s reconstruction accuracy and the continuity of the latent space can be balanced:

(10) $$\begin{array}{c} ELBO=E_{q_{\phi }\left(z|x\right)}\left[\log p_{\theta }\left(x|z\right)\right]-\beta KL\left(q_{\phi }\left(z|x\right)\|p\left(z\right)\right) \end{array}$$

In addition, there are two methods to calculate ELBO: maximum likelihood estimation and stochastic gradient descent (SGD). The disadvantage of the maximum likelihood method is that it is challenging to optimize directly because it usually requires calculating the gradient of the likelihood function for the entire dataset, which is computationally expensive. At the same time, direct sampling from the Gaussian distribution is non-differentiable, which makes the conventional stochastic gradient descent algorithm not directly applicable for training. Using the stochastic gradient descent algorithm will result in the randomness of the sampling process not being captured by the gradient, making the training process difficult to converge. To solve this problem, we propose using the reparameterization trick for stochastic gradient variational estimation. The idea of the reparameterization trick is to separate the sampling process, making the sampling process separate from the differentiable operation, thereby transforming the non-differentiable sampling operation into a differentiable operation so that the conventional stochastic gradient descent algorithm can be directly applied for training. Specifically, the reparameterization trick can introduce the new random variable $e∼N\left(0,I\right)$ and re-express the original random variable z so that z can be rewritten as $z\left(e\right)=\mu_{z}+\sigma_{z}e$, where $\mu_{z}$ and $\sigma_{z}$ are the mean and standard deviation of z. In this way, the sampling process of z(e) is separated from the differentiable operation, can be regarded as a deterministic operation, and can be directly optimized by the stochastic gradient descent algorithm.

In addition, when calculating the KL divergence, there may be numerical instability problems. For example, when the standard deviation is very close to zero, the calculation of logσ may result in an infinite number, which will cause the calculation to be unstable. To solve this problem, we propose to clip logσ and limit it within a smaller range, which we set as $\left[-4,4\right]$. This can avoid unbounded values while retaining enough information to accurately estimate the distance between the latent space and the reconstruction error when calculating the KL divergence.

### 3.6. Anomaly Explanation Based on Reconstruction Probability

In order for the algorithm to provide anomaly explanation capabilities, allowing engineers to locate abnormal devices quickly, we combine the probabilistic theory of VAE and use the reconstruction probability for anomaly explanation. During model training, VAE learns the distribution of the time series. The encoder can compress the time series into latent vectors, and the decoder can generate samples from random vectors in the latent space. Therefore, we use the encoder to obtain the latent vector of each time step and then calculate the reconstruction probability of the sample points in the latent space. In the VAE model, the reconstruction probability can be regarded as a dimension’s contribution to the original data, reflecting the importance of this dimension when the model generates data. This reconstruction probability can be used to calculate the anomaly score of each time step, and these anomaly scores can be used to explain the anomalies in the time series. Specifically, assuming that anomaly data are given at a time step, first use the VAE decoder to generate a reconstruction value ${\hat{x}}_{t}$ as shown in Equation (11):

(11) $$\begin{array}{c} {\hat{x}}_{t}=f_{\theta }\left(Z_{t-T:t}\right) \end{array}$$

where $f_{\theta }$ represents the decoder of VAE, and θ represents the parameters of the decoder. Then, the reconstruction probability $P_{\theta }\left(x_{t}^{i}|Z_{t-T:t}\right)$ of each dimension is calculated, which is the probability of x−t on the *i*-th dimension given the latent variable condition $Z_{t-T:t}$. This can be calculated by comparing the differences between $x_{t}^{i}$ and ${\hat{x}}_{t}^{i}$. Specifically, the Gaussian distribution can be used to assume the probability distribution of $x_{t}^{i}$. Then, the probability density function value of ${\hat{x}}_{t}^{i}$ under this Gaussian distribution is calculated as shown in Equation (12):

(12) $$\begin{array}{c} P_{\theta }\left(x_{t}^{i}|Z_{t-T:t}\right)=N\left(x_{t}^{i}|{\hat{x}}_{t}^{i},\sigma_{i}^{2}\right)=\frac{1}{\sqrt{2\pi \sigma_{i}^{2}}}\exp\left(-\frac{{\left(x_{t}^{i}-{\hat{x}}_{t}^{i}\right)}^{2}}{2\sigma_{i}^{2}}\right) \end{array}$$

where $\sigma_{i}^{2}$ represents the reconstruction error variance on the *i*-th dimension, which the reconstruction error on the *i*-th dimension in the training set can estimate. Normal time series samples can be successfully reconstructed, so their reconstruction probability should be relatively high. They cannot be successfully reconstructed for abnormal time series samples, so their reconstruction probability should be relatively low. To make the anomaly score intuitively display the anomaly situation, the reconstruction probability is transformed using a negative logarithm as shown in the Equation (13):

(13) $$\begin{array}{c} s\left(x_{t}^{i}\right)=-\log P_{\theta }\left(x_{t}^{i}|Z_{t-T:t}\right)=\frac{1}{2}\left(\frac{{\left(x_{t}^{i}-{\hat{x}}_{t}^{i}\right)}^{2}}{2\sigma_{i}^{2}}+\log\left(2\pi \sigma_{i}^{2}\right)\right) \end{array}$$

where $s\left(x_{t}^{i}\right)$ represents the anomaly score of the input sample $x_{t}$ on the *i*-th dimension. The larger the anomaly score, the more abnormal the sample. Since the range of reconstruction probability is $\left[0,1\right]$, negative logarithm transformation can convert it into a non-negative number so that the larger the score, the more abnormal the sample, and the intuitive meaning is retained. Finally, these anomaly scores can be used to explain the contribution of each dimension to the anomaly point; that is, the larger the anomaly score, the more likely that dimension is the main source of the anomaly point.

**Figure 8:**
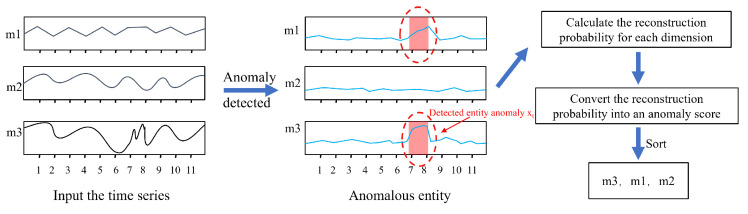
Anomaly interpretation flow chart.

In general, the anomaly explanation can be divided into four steps:

1. For the detected entity anomaly $x_{t}$, calculate the reconstruction probability $P_{\theta }\left(x_{t}^{i}|Z_{t-T:t}\right)$ of each dimension $x_{t}^{i}$, where θ is the parameter of VAE, and $Z_{t-T:t}$ is the latent vector within the time window from t−T to *t*.
2. Convert the reconstruction probability $P_{\theta }\left(x_{t}^{i}|Z_{t-T:t}\right)$ of each dimension into the corresponding anomaly score $S_{t}^{i}$. For this, use the logarithmic transformation: $S_{t}^{i}=-\log\left(P_{\theta }\left(x_{t}^{i}|Z_{t-T:t}\right)\right)$. The logarithmic transformation converts the score into the larger the anomaly score, the more abnormal the sample, which is more convenient for observation.
3. For the detected entity anomaly $x_{t}$, sort all dimensions according to the anomaly score $S_{t}^{i}$ to obtain the sorted list $AS_{t}$. The dimensions in the list are arranged according to their contribution size; the dimensions ranked in the front contribute more to the anomaly of $x_{t}$.
4. Present the sorted list $AS_{t}$ to the operator as an anomaly explanation. The dimensions at the front of the list are more likely to be abnormal devices, allowing the operator to rule out detected entity anomalies quickly. The flowchart of the anomaly explanation is shown in Figure 8.

## 4. Experiments

In this section, we first introduce the dataset and experimental environment, followed by the model parameters. We then experimentally validate the effectiveness of our proposed multi-scale local weight-sharing convolutional neural network model and multi-head attention mechanism. Subsequently, we demonstrate our algorithm’s high accuracy and interpretability through comparative experiments. Finally, we present the results of the ablation experiments.

### 4.1. Dataset and Experimental Environment

SWaT [22] is a small-scale water treatment test platform developed in collaboration with Singapore’s national water company, the Public Utilities Board. It can produce 5 gallons of filtered water per minute, almost perfectly simulating the functionality of real systems. Third-party suppliers manufacture this platform to ensure that its physical processes and control systems are highly similar to actual systems, making the research results applicable to real systems. The industry often uses SWaT to study network attacks and design new industrial control security measures.

The data collection process lasted 11 days, with the system operating 24 h daily, collecting sensor data every second. The system operated normally for the first 7 days, and for the last 4 days, network attacks were carried out under 36 different attack scenarios. The targets of the attacks included physical sensors, actuators, and the network communication infrastructure of CPS. Among them, 28 attacks were focused on a single point, and 8 attacks were simultaneously focused on multiple points. Researchers sometimes carried out attacks in sequence and sometimes allowed the system to recover to normal before carrying out the next attack. The attack phase of this dataset spanned from 4:30 PM on 22 December 2015, to 10:00 AM on 28 December 2015, covering approximately 3 days. All 51 sensors sampled data every second, resulting in 495,000 s of data. The data input format is a CSV file containing 495,001 rows of records, with each row capturing a timestamp and the corresponding 51 sensor and actuator measurements. During model training, we employed a sliding window of size 30, where each input sample comprised data from 30 consecutive time points. Each time point included 51 feature values, resulting in a dimension of 30 × 51 for each input sample.

In terms of software, we used tensorflow-gpu version 1.12.0, CUDA 10.0, Python version 3.8, and the operating system was ubuntu16.04. In terms of hardware, our server configuration was as follows: The GPU is RTX 2080 (11 GB) (manufactured by NVIDIA Corporation, located in Santa Clara, CA, USA), the CPU is Intel^®^ Xeon^®^ Platinum 8255C CPU @ 2.50 GHz (manufactured by Intel Corporation, located in Santa Clara, CA, USA), and the memory is 43 GB.

### 4.2. Operating Parameter Settings and Comparison Models

The hyperparameters used in the experiment are shown in Table 1.

We selected three baseline algorithms and reproduced their codes. DAGMM [13] combines the reconstruction error from an autoencoder with density estimation from a Gaussian mixture model (GMM). By jointly optimizing these components in an end-to-end fashion, DAGMM effectively avoids local optima while significantly improving anomaly detection performance for high-dimensional data, all while preserving essential information. LSTM-NDT [24] proposes a self-supervised framework that treats each univariate time series as a separate feature and includes two parallel graph attention layers to capture the relationships between different time series effectively. TDAD [25] introduces a transformer with a discriminator for high-precision multi-variate time series detection, incorporating adversarial training to improve loss handling and attention mechanism-based feature extraction.

### 4.3. Effectiveness of MCA-VAE

**Figure 9:**
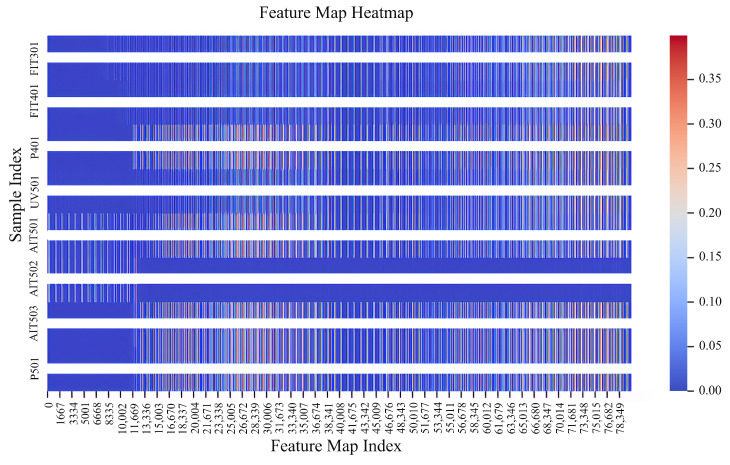
Feature heatmap.

First, we evaluate the effectiveness of our multi-scale weight-sharing network in extracting temporal features between dimensions. We select 8 sensors: FIT301, FIT401, P401, UV401, AIT501, AIT502, AIT503, P501. Then, we merge their data into a two-dimensional dataset and use the multi-scale weight-sharing convolutional network for feature extraction. We then draw the feature heatmap as shown in Figure 9.

In the feature heatmap, the horizontal axis represents the feature values, and the vertical axis represents the device number. Flattening all feature maps, each 1667 activation value is taken as a group and displayed as a square in the heatmap. The color represents the size of the activation value. The brighter the area, the larger the group of activation values, so the importance of this feature at this location is higher. In the feature heatmap, many features have high activation values in a specific interval, indicating that this feature is very important for distinguishing samples. Observing the feature heatmap, you can see that the color of UV501 is the brightest, and the color of AIT502 is lighter. Combined with the dataset, it can be found that UV501 is the core sensor; its value will greatly affect the operation of the entire system, the data change amplitude and frequency of the UV501 dimension are huge, and more time series features can be obtained. The role of AIT502 is relatively small, the data change smoothly, with fewer time series features, and the performance of the feature map is consistent with the dataset analysis. Therefore, our multi-scale, multi-channel convolution kernel can effectively extract time series features within the dimension from the dataset.

**Figure 10:**
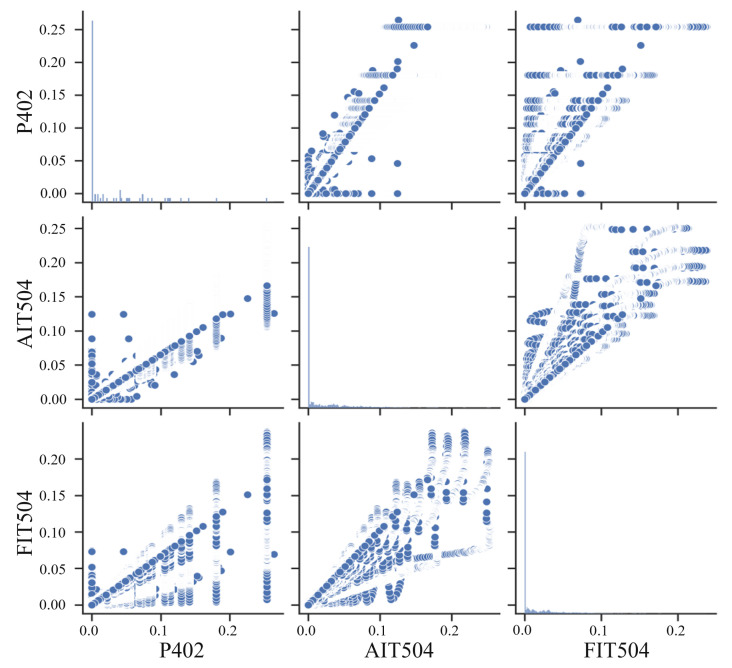
Feature relationship diagram.

Next, we evaluate the effectiveness of the multi-head attention mechanism module in extracting the correlation between dimensions. We select three sensors with strong correlation, FIT504, AIT504, and P402, use the multi-head attention mechanism to extract correlation features, and then draw the feature relationship diagram as shown in Figure 10.

The horizontal axis represents a group of features, and the vertical axis represents another group of features. Each feature index is combined with other indexes to display their relationship; each scatter plot represents the correlation between a pair of features. The figures on the diagonal line are the distribution of each feature, and the figures off the diagonal line are the distribution relationship between features. From the figure, we can intuitively see the relationship between features. The figures of the diagonal line are apparent, and there is a strong clustering phenomenon. They are generally distributed near a linear correlation, indicating that our model has successfully extracted the correlation features.

**Figure 11:**
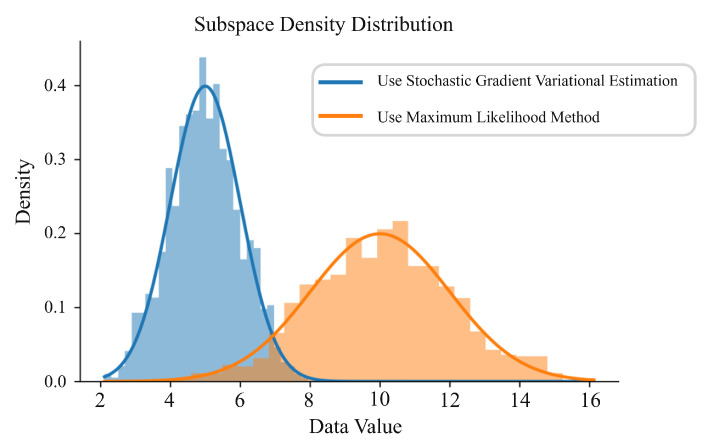
Density distribution of latent space.

We select the values of the UV501 sensor for training and plot the latent space density distribution curve as shown in Figure 11. The figure shows that the stochastic gradient variational estimation method is closer to the normal distribution. This is because training with Stochastic Gradient Variational Bayes (SGVB) can better approximate the latent space distribution of the generated data distribution, thereby better learning the data’s distribution structure. The generated samples will be more continuous and natural, making the VAE more accurate when generating new samples and having better generalization performance.

### 4.4. Experimental Results and Analysis

**Figure 12:**
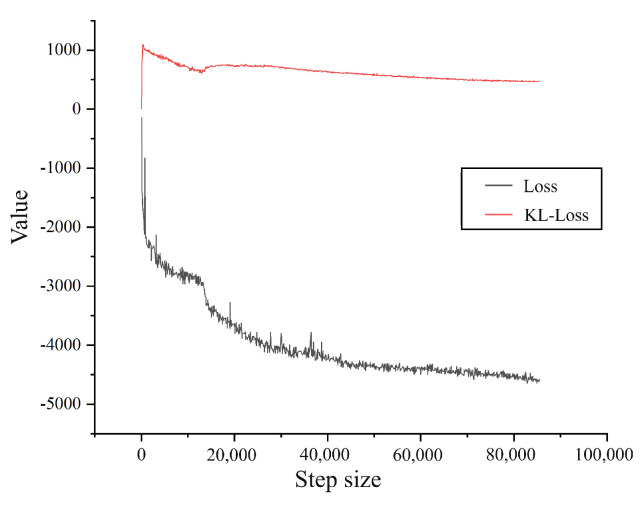
Loss function.

The model training diagram is shown in Figure 12, which includes the overall loss function (Loss) and the KL loss function (KL_LOSS). We first analyze the trend of the loss function in the figure. It can be seen that the loss value decreases rapidly in the early stage of training, indicating that the model is learning the features of the data. At step 80,000, the loss value is stable, indicating that the model has basically converged. During the training process, we use the negative ELBO (SGVB). Our goal during training is to maximize the ELBO, which means we are minimizing the negative ELBO (SGVB). In Figure 12, the red curve represents the KL loss, while the black curve represents the overall loss. The red curve shows that the KL loss is positive. KL divergence measures the difference between the posterior distribution $q_{\phi }\left(z|X\right)$ and the prior distribution $p\left(z\right)$, and it is always greater than or equal to zero. The loss is negative and decreases over time, aligning with our expectations.

**Figure 13:**
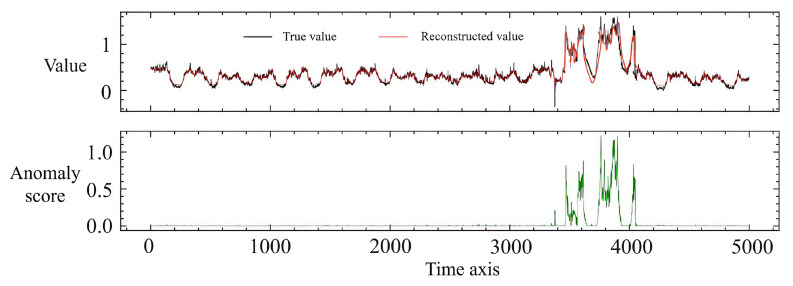
Anomaly detection effect diagram.

To display the model detection effect more intuitively, we select the data from the dataset between 09:50:00 on 28 December 2015 and 10:50:00 on 28 December 2015. This is because the attack at this stage is multi-point, which causes a heavy blow to the system and has a significant impact area. This process involves both the chain reaction of a single sensor affecting the values of subsequent sensors and the impact of parameter tampering targeting multiple sensors. Therefore, the anomaly detection at this stage can better demonstrate our detection results as shown in Figure 13.

The upper part of the figure shows the original data values and the reconstructed data values, where the original data are colored black, and the reconstructed data are red. The two lines basically overlap. The lower part of the figure is the anomaly score calculated by the model. It can be seen that where the original values in the upper figure fluctuate dramatically, the lower figure corresponds well, indicating that our model’s detection effect at this stage is very good.

**Figure 14:**
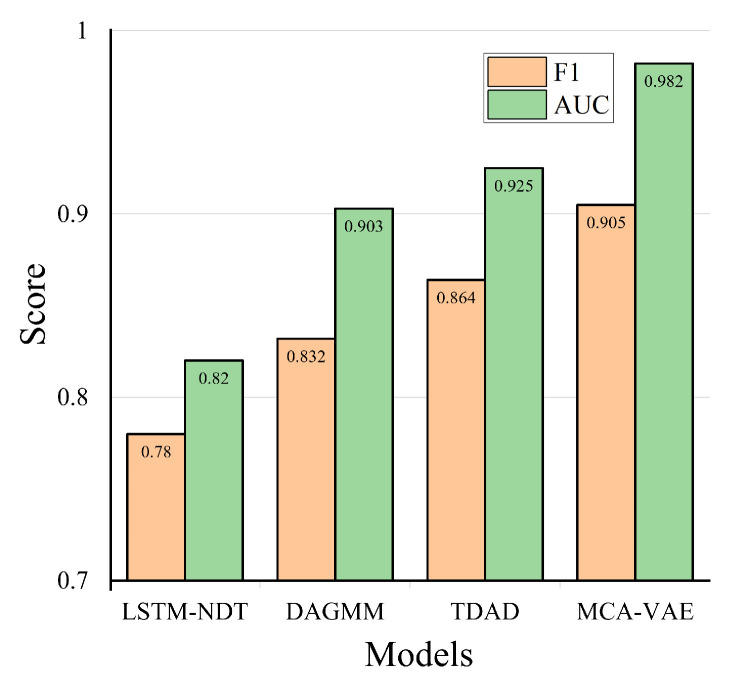
Model performance comparison chart.

Next, to more comprehensively evaluate the performance of our model and similar research results, we conduct five repeated experiments for each method, select the highest score among them, and draw a model performance comparison chart as shown in Figure 14. As can be seen from the F1 and AUC overall indicators, MCA-VAE is superior to all baselines, and compared with the second-best algorithm TDAD, our model’s F1 has increased by 4%. Our model proposes an improved multi-scale weight-sharing convolutional network to extract temporal features, a multi-head attention mechanism to extract inter-dimensional features, and an enhanced variational autoencoder that can extract more features, improving the accuracy of subsequent anomaly detection tasks. Other methods mainly model one type of dependency in MTS. For example, LSTM-NDT models time dependency, while DAGMM, and TDAD focus on inter-dimensional correlation. Among them, LSTM-NDT shows the lowest performance, the reason being that it only models temporal features. The inter-dimensional feature relationships between SWaT data are close, so DAGMM and TDAD achieve good results. Among them, the effect of TDAD is second best because it uses an attention mechanism to extract inter-dimensional features further, improving the detection effect.

We compare the training time of our model with the baseline models as shown in Table 2. It can be seen that MCA-VAE significantly reduces the training time compared to the baseline algorithms. This is because our local weight sharing reduces the number of parameters, thereby speeding up the training. LSTM-NDT also models each time dimension, but the LSTM model is more time-consuming, leading to slow training. The DAGMM network may have the longest training time because it needs to train two modules: the compression and estimation networks.

**Figure 15:**
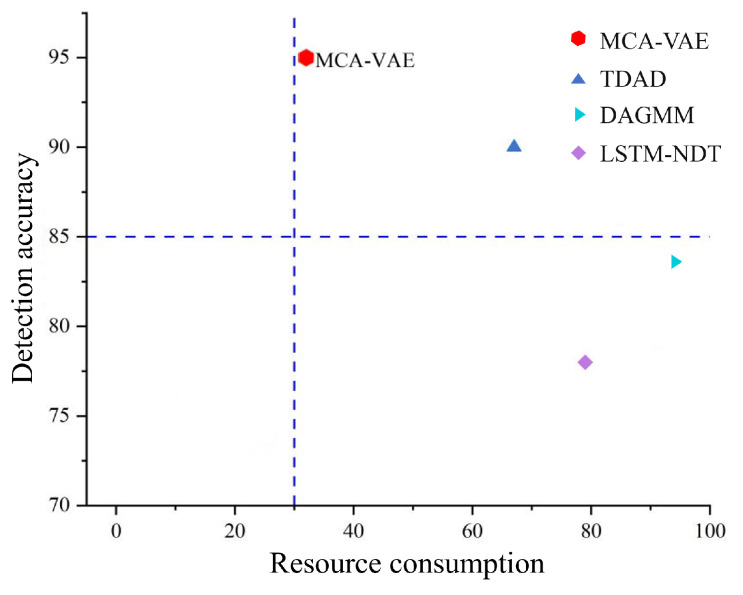
Comprehensive performance and resource comparison chart.

We take the weighted values of memory consumption and time consumption as the x-axis and the weighted values of the F1 score and AUC score multiplied by 100 as the y-axis to draw a comprehensive performance and resource consumption figure as shown in Figure 15. We define the boundary for resource consumption as a weighted value of 30 for memory and time consumption; models with values greater than 30 are considered complex models, while those with values less than 30 are deemed lightweight models. The performance indicator 85 is used as the boundary; models with values greater than 85 are high-precision models, and those with values less than 85 are general precision models. MCA-VAE leads other baseline models in detection accuracy and is close to lightweight models regarding resource consumption. The experimental results prove that our algorithm significantly improves performance and resource consumption compared to similar algorithms.

### 4.5. Anomaly Interpretation Effect

**Figure 16:**
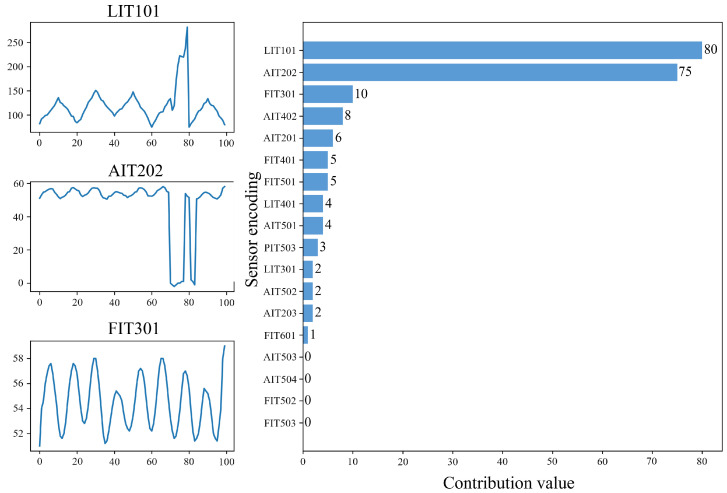
Anomaly interpretation effect.

To better demonstrate the anomaly explanation module we propose, we select three representative sensors out of 51 and capture a period of their records. These three sensors are simultaneously subjected to multi-point attacks during this period as shown in Figure 16.

Observing the original data in the left figure, it can be seen that the LIT101 sensor exhibits anomalies within the time dimension. By observing the historical data, it can be seen that its data will linearly increase or decrease within 10 data points, complete an alternating cycle, and return to the origin within 20 data points. However, between 70 and 80 data points where the sensor was attacked, its value shows violent fluctuations, which is inconsistent with its periodicity. The AIT202 sensor exhibits inter-dimensional anomalies. Observing the values of the FIT301 and AIT202 sensors, it can be found that the values of AIT202 and FIT301 are positively correlated; that is, they rise and fall together. Between 70 and 80 data points where it was attacked, the value of AIT202 shows an irregular trend, which is inconsistent with the periodicity between dimensions.

The right figure is a bar chart of anomaly scores generated after calculating the reconstruction probability according to our anomaly explanation algorithm and taking the negative logarithm change. The data in the figure show that the anomaly scores of LIT101 and AIT202 are significantly higher than other sensors during the anomaly detection stage. Although FIT301 was also attacked, the impact on this sensor is relatively small, so the anomaly score is significantly lower than LIT101 and AIT202. The scores of other unaffected sensors have a large gap with these three, so this result can effectively indicate to technicians to troubleshoot anomalies quickly.

### 4.6. Ablation Experiments

**Figure 17:**
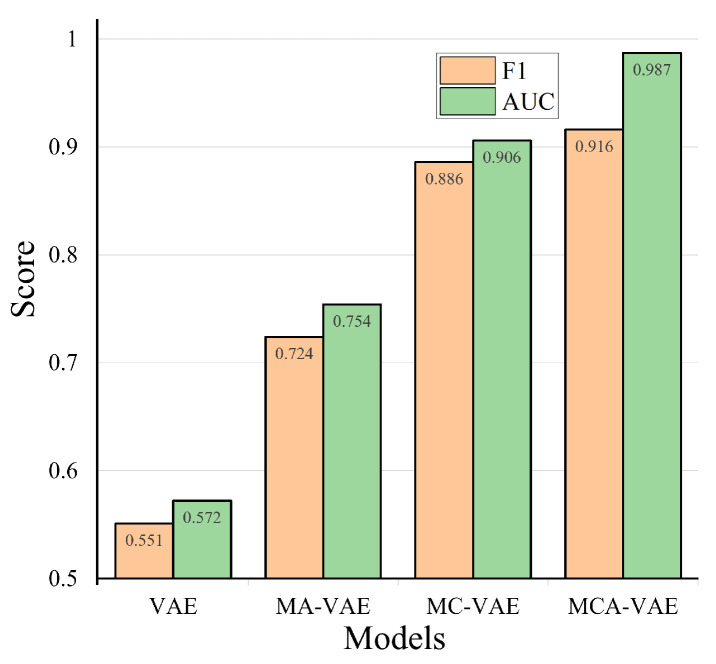
Effect diagram of ablation experiment.

We conduct comparative experiments on different model components to demonstrate the necessity of each module in our algorithm. We separately use the standalone VAE model, the MC-VAE model, which uses a multi-scale weight-sharing convolutional neural network; the MA-VAE model, which uses an attention mechanism and a variational autoencoder, and MCA-VAE for ablation experiments. The experiments are conducted thrice, and the best results are selected as shown in Figure 17.

MCA-VAE achieves the best results because the model can simultaneously extract temporal and inter-dimensional features. Analyzing the comparative experimental results, it can be found that the effect of using VAE alone for anomaly detection is the worst because it is difficult to find the correlation between dimensions by only using reconstruction errors. The MA-VAE model, which adds a multi-head attention mechanism before the VAE model, greatly improves the effect because the attention mechanism can effectively extract the relationship features between variables before the original multi-dimensional data are mapped to latent variables. The MC-VAE model, which performs convolution operations for each dimension, achieves better results. The analysis is because the time features within the dimension in the SWaT dataset account for a larger proportion, so this model can also achieve good results.

## 5. Conclusions

We analyze two types of features in industrial control system: intra-dimensional temporal features and inter-dimensional relationship features. Given that the current model cannot effectively extract these two features simultaneously and lacks interpretability, we propose a high-precision interpretable anomaly detection algorithm based on VAE, MCA-VAE. We first propose a multi-scale weight-sharing CNN to extract temporal dependency features and then use a multi-head attention mechanism to extract inter-dimensional correlation features. To address the convergence problem of KL divergence and the issue of non-interpretability, we propose an improved VAE and use a reconstruction probability model for anomaly explanation. Experimental results show that our algorithm achieves an AUC value of 0.982 and an F1 score of 0.905 in anomaly detection, which is 4% higher than the best-performing baseline algorithm, TDAD. It also provides accurate anomaly interpretation capability. In the future, we can introduce more features, such as spatial dimensional features, to reflect the system’s operating status more comprehensively.

## Figures and Tables

**Table 1:** Hyperparameters of MCA-VAE.

Parameter	Value	Description
Window length	30	Sliding Window Size
Conv 1D hidden layers	3	CNN Hidden Layers
num_heads	8	Attention Head Count
key_dim, query_dim, value_dim	64	Dimension of Attention Query, Key, and Value Vectors
latent_dim	10	VAE Latent Variable Dimension
Batch Size	10	Control the Number of Samples Drawn in Each Training Process

**Table 2:** Model training time.

Models	Training Time (s)
TDAD	289
LSTM-NDT	609
DAGMM	1042
**MCA-VAE**	**95**

## Data Availability

The data used to support the findings of this study are available from the corresponding author upon request.
